# Smegma Pearl in a Circumcised Patient: A Case Report

**DOI:** 10.7759/cureus.28491

**Published:** 2022-08-28

**Authors:** Yaser Mansoor Almutawa, Aneesh Bava, Shana Shirin Najeeb

**Affiliations:** 1 Dermatology and Venerology, King Hamad University Hospital, Busaiteen, BHR; 2 Dermatology and Venerology, Salmaniya Medical Complex, Manama, BHR; 3 Public Health, Amrita Institute of Medical Sciences & Research Center, Kochi, IND

**Keywords:** comedone extractor, penile cyst, foreskin, circumcision, smegma pearl

## Abstract

Smegma pearls, also called smegmomas, are a natural secretion composed of epithelial cells, protein, and fat. Smegma pearls are not carcinogenic and do not induce damage or irritation. Despite the frequent occurrence of smegma pearls in uncircumcised children, no case among circumcised patients was reported. Here, we are presenting, for the first time, a case of smegma pearl in a circumcised patient. A 36-year-old circumcised male patient presented with a smegma pearl. The purpose of the present case report is to describe the diagnosis of this benign condition in circumcised patients, yet not described in the literature, and help physicians to enhance patient management and avoid needless investigations or treatments.

## Introduction

The smegma appears as accumulations of opaque-white or yellow substance composed of dead cells and skin oils, which is malodorous [1]. It constitutes the emission of the sebaceous gland that accumulates between the foreskin and the glans [2]. This can get entrapped and transform into a lump or nodule named smegma pearl [3]. Smegma pearls are described as a benign group of smegma in the sub-preputial surface of uncircumcised individuals. Despite being common, they have been reported only recently [4].

Smegma pearls do not present covering sac, while smegma cysts are characterized by a well-formed epithelial wall. Untreated, smegma pearls can be transformed into a smegmolith, a solid stone-like form, owing to mineral accumulation and persistent irritation [5]. This condition happens before the retraction of the foreskin in children or if the prepuce is not retracted regularly to clean the glans in older boys [6]. Smegma pearls may produce a visible swelling, which may be misdiagnosed as a tumor or dermoid cyst. Generally, they spontaneously resolve over time as the foreskin separates from the glans, and this may induce some redness and irritation of the foreskin [7]. Smegma pearls are not infectious, and proper hygiene is the unique required treatment as the prepuce becomes more retractile [8]. Oster et al. reported that this condition was observed in 5% of healthy children [9]. To the best of our knowledge, based on a thorough review of the literature using Medline, PubMed, and Google Scholar, no studies have reported a case of smegma pearls in a circumcised patient.

## Case presentation

**Figure 1:**
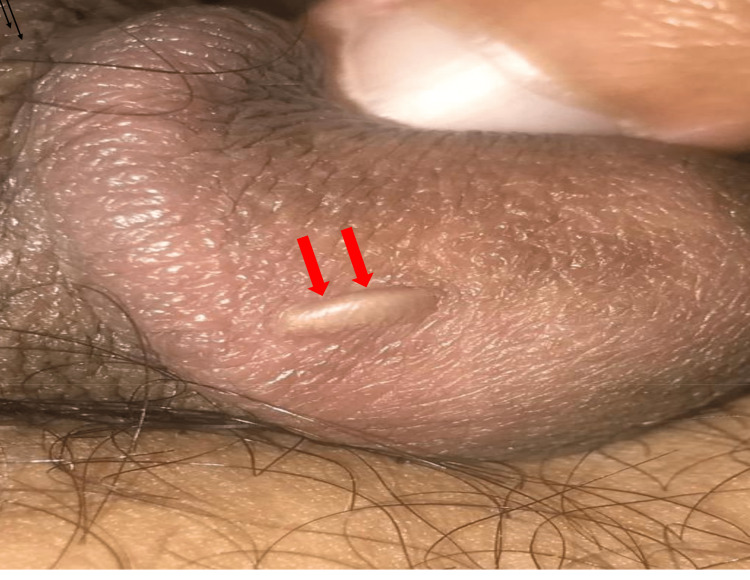
Smegma pearl/keratin pearl in the genitalia (red arrows)

A healthy 36-year-old circumcised male patient presented to the dermatology clinic with a painless whitish-yellow pearly nodule over the glans that appeared one year before the presentation. There were no symptoms associated with the lesion, nor was he in pain or discomfort, and he denied having suffered any traumatic injury or urinary symptoms. Aside from his lesion on his genitalia, he had no lesions anywhere else on his body and he had relatively normal vital parameters. The medical history of this patient did not play a part in the apparition of this nodule. Similarly, there was no family history related to this condition. Other than the lump on his penis, the physical examination was normal. The nodule was located below the foreskin on the right face. The size of the nodule was 0.5 X 2 cm with an oval shape and it was along the axis of the genital organ (Figure 1).

**Figure 2:**
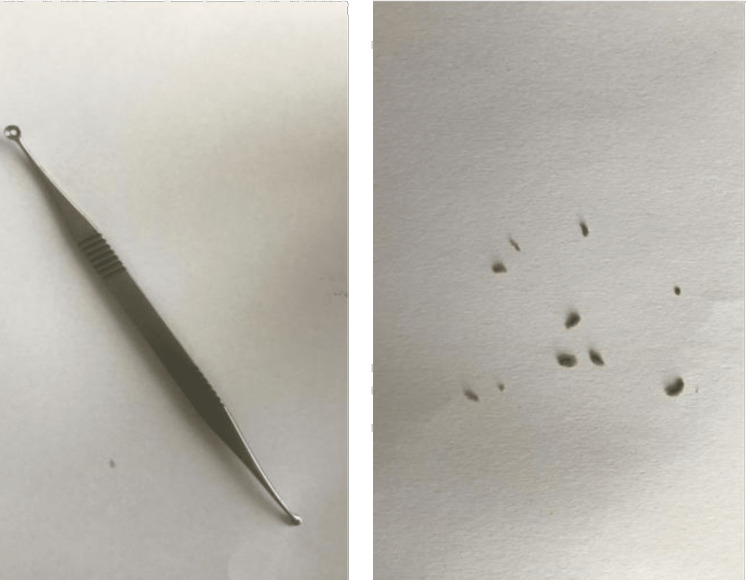
Smegma extruded out with comedone extractor

Application of gentle pressure with fingers on nodule material could not extrude it. Hence, the patient was placed in the supine position, topical anesthesia was given, the wire loop of the comedone extractor was fixed on the nodule, and gentle pressure was applicated to the nodule releasing odorless cheese-like whitish material (Figure 2). The patient was seen one month later, when he stated that he was doing well, and the lesion was gone completely. Upon physical examination, no new lesions were noticed.

## Discussion

In this report, we described a nodule of entrapped smegma, termed “smegma pearl/keratin pearl”, which has been reported in uncircumcised boys. Our case is the first reported in a circumcised male. The development and role of smegma pearl and its clinical features have been well-clarified. It is a benign collection of smegma in sub-preputial space. Smegma has a typical slimy odor, consisting of epithelial debris, fat, and proteins [10]. The smegma pearl is a yellowish nodule that is frequently detected on the ventral side of the glans in uncircumcised individuals. It is seen between the base of the glans penis and the sub-preputial surface. The prepuce is partially retractable and the urethra is not infected [5]. In 50% of cases, smegma pearl presents a varied bacterial flora and a bacterial superinfection with smegma bacillus can occur. However, Howe et al. have reported that smegma pearl is neither an irritating nor damaging lesion and is not carcinogenic also [11]. Sometimes, smegma pearls are misdiagnosed as abnormal growth and physicians require surgical excision, which is not necessary [12].

Apart from smegma pearls, the differential diagnosis of pearly lesions could include preputial Epstein pearls, preputial cysts, and preputial median raphe cysts, which are easily clinically discernible [6,10]. The location and age of onset help distinguish these from smegma pearls. Preputial Epstein pearls, a benign condition, are composed of keratinized epithelial cells during the growth of the fetus. They are the homolog of Epstein pears in newborns. They are located at the tip of the foreskin and fade after one week of life. Preputial cysts also called keratin pearls, arise from preputial skin, and occur due to the collection of dead skin. They are identified as nodular swelling and are located under the glans [13]. However, median raphe cysts are situated near the glans. It was reported that the development of median raphe cysts could be due to a problem in the closure of the urethra that occurred during the development of the embryo [14]. Penile pearls are tiny and flexible nodules. Generally, they are asymptomatic but they can present obstruction or infection. They spontaneously exfoliate, and management is mainly reassurance [15].

To date, the detailed physiological mechanism underlying the development of these cysts in some boys remains unknown. Similarly, it is still unclear what factors influence the development of smegma pearl in uncircumcised boys rather than in circumcised ones. Unfortunately, information related to smegma pearls and their anatomic basis in the extant literature was limited.

The treatment of smegma pearl could include bimanual retraction of smegma but this can cause paraphimosis. It can also induce several side effects including recurrence of phimosis and smegma pearl, as well as the infection of the foreskin [16]. Hence, all that is required is reassurance for spontaneous separation to take place over time.

## Conclusions

Smegma pearls have a natural composition of dead cells and skin oils between the foreskin and the glans. They are benign and they spontaneously resolve over time. Good personal hygiene is sufficient to control smegma pearls. The present work gives further insight into the diagnosis of this condition in circumcised men, yet not reported previously, and helps physicians to improve the treatment and avoid useless interventions. The extraction of smegma pearl using a comedone extractor is a simple, safe, and cheap procedure. However, potential complications of manual expression of smegma out of them could occur. Hence, the best approach is to leave them to resolve naturally over time.
